# Satellite-Based Estimation of Hourly PM_2.5_ Concentrations Using a Vertical-Humidity Correction Method from Himawari-AOD in Hebei

**DOI:** 10.3390/s18103456

**Published:** 2018-10-14

**Authors:** Qiaolin Zeng, Liangfu Chen, Hao Zhu, Zifeng Wang, Xinhui Wang, Liang Zhang, Tianyu Gu, Guiyan Zhu, Yang Zhang

**Affiliations:** 1 State Key Laboratory of Remote Sensing Science, Institute of Remote Sensing and Digital Earth of Chinese Academy of Sciences, Beijing 100101, China; zengql@radi.ac.cn (Q.Z.); Chenlf@radi.ac.cn (L.C.); wangzf@radi.ac.cn (Z.W.); 2 University of Chinese Academy of Sciences, Beijing 100049, China; 3 Chongqing Institute of Meteorological Sciences, Chongqing 401147, China; 4 Remote Sensing Monitoring, Beijing Municipal Environmental Monitoring Center, Beijing 100048, China; saint.tail.always@163.com; 5 Environmental Emergency and Heavy Pollution Weather Warning Center, Shijiazhuang 050051, China; gty0707@163.com (T.G.); 18931896527@163.com (G.Z.); zhangyang89109320@163.com (Y.Z.)

**Keywords:** satellite remote sensing, PM_2.5_, vertical and RH correction, Hebei

## Abstract

Particulate matter with an aerodynamic diameter less than 2.5 μm (PM_2.5_) is related to various adverse health effects. Ground measurements can yield highly accurate PM_2.5_ concentrations but have certain limitations in the discussion of spatial-temporal variations in PM_2.5_. Satellite remote sensing can obtain continuous and long-term coverage data, and many previous studies have demonstrated the relationship between PM_2.5_ and AOD (aerosol optical depth) from theoretical analysis and observation. In this study, a new aerosol product with a high spatial-temporal resolution retrieved from the AHI (the Advance Himawari Imager) was obtained using a vertical-humidity correction method to estimate hourly PM_2.5_ concentrations in Hebei. The hygroscopic growth factor (f(RH)) was fitted at each site (in a total of 137 matched sites). Meanwhile, assuming that there was little change in f(RH) at a certain scale, the nearest f(RH) of each pixel was determined to calculate PM_2.5_ concentrations. Compared to the correlation between AOD and PM_2.5_, the relationship between the “dry” mass extinction efficiency obtained by vertical-humidity correction and the ground-measured PM_2.5_ significantly improved, with r coefficient values increasing from 0.19–0.47 to 0.61–0.76. The satellite-estimated hourly PM_2.5_ concentrations were consistent with the ground-measured PM_2.5_, with a high r (0.8 ± 0.07) and a low RMSE (root mean square error, 30.4 ± 5.5 μg/m^3^) values, and the accuracy in the afternoon (13:00–16:00) was higher than that in the morning (09:00–12:00). Meanwhile, in a comparison of the daily average PM_2.5_ concentrations of 11 sites from different cities, the r values were approximately 0.91 ± 0.03, and the RMSEs were between 13.94 and 31.44 μg/m^3^. Lastly, pollution processes were analyzed, and the analysis indicated that the high spatial-temporal resolution of the PM_2.5_ data could continuously and intuitively reflect the characteristics of regional pollutants (such as diffusion and accumulation), which is of great significance for the assessment of regional air quality.

## 1. Introduction

Many researchers have demonstrated that particulate matter with an aerodynamic diameter less than 2.5 μm (PM_2.5_) is related to various adverse health effects, such as respiratory mortality, lung diseases, and cardiovascular disease [1,2,3,4,5]. With the rapid development of industrialization and urbanization, PM_2.5_ increasingly leads to terrible air quality and has been a hot research topic for public health. Donkelaar et al. [2] suggested that China is one of the most important regions in the world with respect to air pollutants; thus, many environmental monitoring stations have been built in China since 2013. Ground-based stations can measure PM_2.5_ concentrations and compositions with a relatively high accuracy. However, these stations are mainly located in cities, and the distribution of stations is sparse and asymmetrical; therefore, research on the spatial-temporal variation in PM_2.5_ has certainly limitations [6]. Conversely, satellite remote sensing can obtain seamless and long-term coverage data, and the aerosol optical depth (AOD) of retrieval has been widely used to predict PM_2.5_ concentrations [7,8,9,10,11,12]. AOD is the column integration of light extinction (scattering and absorption) in the atmosphere and is relative to the physicochemical properties of particles (e.g., radius, composition, and refraction index). Many previous studies have demonstrated the relationship between PM_2.5_ and AOD from theoretical analysis and observation. Methods for demonstrating this relationship are mainly classified into two categories: observation-based methods and simulation-based methods [9]. Observation-based methods include the proportional factor method [13,14,15], semi-empirical formula method [16,17,18], and statistical model method [8,19,20,21,22], which rely on ground-measured and meteorological parameters and have a relatively high PM_2.5_ estimation accuracy, while observation-based methods do not consider the effect of chemical composition. The effects of meteorology and particle properties are considered in simulation-based methods at global or regional scales, but these methods have some uncertainties (e.g., emission uncertainties) that can lead to inaccurate results [23,24,25].

The correlation between AOD and PM_2.5_ is highly influenced by the vertical distribution of AOD and the relative humidity (RH). These two parameters are concerned with atmospheric profiles, ambient conditions, and aerosol sizes, which might have large spatiotemporal variations. Many scholars have studied physicochemical impacts on the AOD-PM relationship and have improved the accuracy of PM_2.5_ estimation. Li et al. [6] indicated that the correlation between AOD and PM_10_ was improved by up to 0.54 after being corrected by the vertical-and-RH correction method. Koelemeijer et al. [26] demonstrated that this scaling of the AOD with planetary boundary layer height (PBLH) and RH improved the time-correlation with PM_2.5_ (r = 0.6). Guo et al. [27] studied the correlation between RH-corrected AOD and PM_2.5_ in Eastern China in 2007 and found a higher correlation with hourly average PM_2.5_ concentrations (r = 0.61) and daily average PM_2.5_ concentrations (r = 0.58). Wang et al. [28] used the vertical-humidity correcting method to estimate the PM in Beijing, with R^2^ increasing from 0.35 to 0.56 (from 0.35 to 0.66) for PM_2.5_ after vertical (RH) correction. Wang et al. [10] collected visibility (VIS), RH and PM_10_ data to discuss the impact of RH correction on PM_10_ estimation and suggested that the monthly correlation between aerosol extinction coefficients and PM_10_ increased from 0.26–0.63 to 0.49–0.82 after RH correction. Lin et al. [9] considered the effects of aerosol characteristics (aerosol composition and size distribution) to quantify the PM_2.5_ distribution in Eastern China, and this consideration improved the correlation between satellite-estimated and ground-measured annual and monthly PM_2.5_ averages, with r values of 0.90 and 0.76, respectively. He et al. [29] analyzed the effect of RH in East China and concluded that higher hygroscopic growth regions can relate to more sulfates and nitrates, and the correlation between satellite estimations and ground measurements was more than 0.85.

However, most studies have obtained a limited number of ground-measured PM_2.5_ data and have developed correlative linear models between AOD and PM_2.5_ to estimate regional PM_2.5_ concentrations with insufficient accuracy, and few studies have fit the hygroscopic growth function at each ground-measured site. Furthermore, the temporal resolution of these studies is relatively low, which prevents the adequate monitoring of the spread and accumulation of pollutants. Due to economic development, Hebei province is the most polluted area in China, and more dense sites are being established. A geostationary satellite (Himawari-8) has provided hourly resolution data since 2015, making it feasible to estimate hourly PM_2.5_ concentrations with relatively high accuracy and robust validation. This study proposes a new vertical-RH correcting method to estimate hourly PM_2.5_ concentration in Hebei province by fitting the hygroscopic growth function at each ground-measured site, which both temporal and spatial resolution are improved compared with previous studies. This paper is structured as follows: descriptions of the observational dataset are reported in Section 2; Section 3 describes the theoretical basis of the vertical-RH correcting method and proposes an equation for PM_2.5_ estimation. Section 4 presents the hygroscopic model fitting results and evaluates the estimated PM_2.5_ accuracy at different time scales and stations. Finally, the conclusions are given. 

## 2. Study Area and Data

### 2.1. Study Area

**Figure 1:**
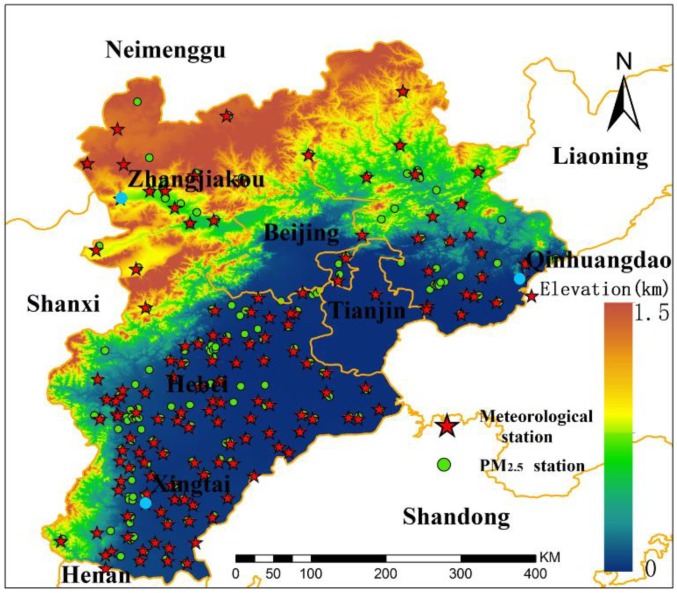
Study region with environmental monitoring stations and meteorological stations.

The study area is Hebei province, which has a spatial extent of 36°05′ to 42°40′ N latitude and 113°27′ to 119°50′ E longitude (Figure 1). Hebei is the only province in China with highlands, mountains, hills, basins, plains, grassland, and seashores and has a total area of 188,800 km^2^ and a permanent population of approximately 75 million. Hebei includes 11 cities, and the northernmost part of the province belongs to the Mongolian Plateau, with high altitudes; the southern part of the province comprises plains with a low altitude. Hebei has a temperate monsoon climate and is dry with little rain in winter. The air pressure is low, and the air does not flow. Hebei has important steel and coal sites in the north. The unfavorable climatic conditions and massive pollutant emissions of this province lead to poor air quality, so Hebei is one of the most polluted provinces in the China.

### 2.2. Data

#### 2.2.1. AHI AOD

Himawari-8, a geostationary satellite launched by the Japan Meteorology Agency (JMA) on 7 October 2014, carries the primary instrument of the Advance Himawari Imager (AHI). AHI is a 16-channel multispectral imager with wavelengths spanning a range from 0.47 to 13.3 μm, including 3 visible (VIS), 3 near-infrared (NIR), and 10 infrared (IR) bands. The imager has the highest spatial resolution at 0.5 km and provides observations approximately every 30 min over China. In this study, the AHI_AOD, which were retrieved by the method of Yang et al. [30], provided AOD data. They used the AHI and Moderate Resolution Imaging Spectroradiometer (MODIS) spectral response functions to make the relationship more suitable for AHI, and a new dark target algorithm was proposed to retrieve the AOD at 1 km resolution over Mainland China. Yang et al. downloaded the Himawari-8 level three hourly AOD (AOD_JAXA) data from the Japan Aerospace Exploration Agency (JAXA) for comparison with their retrieval results (http://www.eorc.jaxa.jp/ptree/index.html), and extracted satellite data and Aerosol Robotic NETwork (AERONET) data from 02:00 to 07:00 (UTC). Except for that at 02:00 (UTC), the R^2^ of AHI_AOD is higher than that of AOD_JAXA. Meanwhile, seasonal averages showed that their product is more similar to MODIS Collection 6 (C6) Dark Target (DT) [31] AOD than AOD_JAXA.

#### 2.2.2. Meteorological Data

A total of 142 meteorological sites, with data including visibility (VIS), RH, and wind direction, were obtained from the Hebei Province Meteorological Bureau. The temporal resolution was 1 h from January to June 2017, which can be matched with the AHI AOD and PM_2.5_ data. To reduce errors, visibility data were omitted when the daily average visibility was less than 1/3 of the values in the next and previous days [32].

#### 2.2.3. PM_2.5_ Data

The ground-level PM_2.5_ observations over Hebei from January to June 2017 were obtained from the Hebei Province Environmental Monitoring Center and had a temporal resolution of 1 h. PM_2.5_ concentrations are measured using the tapered element oscillating microbalance (TEOM) approach or beta-attenuation approach, both of which comply with the National Standard for Environmental Air Quality (GB3095-2012) [33]. The PM_2.5_ data need be somewhat regular to avoid affecting the fitting results of aerosol hygroscopic growth. (1) PM_2.5_ levels less than the 3rd percentile or more than the 97th percentile within 3 h were not used for calculations. (2) Data with humidity less than 70% when the extinction coefficients exceeded the 80th percentile were omitted. (3) The distances between pairs of meteorological sites and environmental monitoring sites had to be within 10 km; therefore, there were 198 PM_2.5_ observation sites that could be matched with 137 meteorological sites.

## 3. Methodology

AOD is the integration of the extinction coefficients absorbed and scattered by aerosols in an atmospheric column; thus, to obtain the surface aerosol extinction coefficient from AOD, vertical correction is needed. The physicochemical characteristics of an aerosol particle are changed because of absorbing or evaporating water vapor in the atmosphere, so humidity correction is needed to obtain the “dry” aerosol extinction coefficient.

### 3.1. Vertical Correction

By assuming that the plane atmosphere is homogeneous, AOD is the integral of the extinction coefficient ($\sigma_{a}$) at all altitudes along the vertical orientation [34]. Assuming the vertical distribution of $\sigma_{a}$ is the negative exponent form, AOD can be expressed by Equation (1) [9,28,35,36]

(1) $$\mathrm{AOD}=\int_{0}^{\infty }\sigma_{a,0}\cdot e^{-z/H}\mathrm{dz}=\sigma_{a,0}\cdot H$$

where $\sigma_{a,0}$ stands for the surface aerosol extinction coefficient at the wavelength of 550 μm, Z is the vertical height, and H is the scale height of aerosols. H can be approximately replaced by the boundary layer height [26,37]. Koschmieder [38] assumed that the impact of air molecules can be neglected when the threshold contrast of human eyes takes the common value of 0.02; thus, the $\sigma_{a}\left(\lambda \right)$ can be expressed as

(2) $$\sigma_{a}\left(\lambda \right)=\frac{3.912}{\mathrm{VIS}}-\frac{32\pi^{3}{\left(n-1\right)}^{2}}{3{N\lambda }^{4}}$$

where VIS is visibility, and n and N represent the atmospheric refractive index and the number density of molecules (n − 1 = 293 × 10^−6^ and N = 266 × 10^19^ at the sea level), respectively. λ stands for wavelength. Thus, H can be calculated by AOD and VIS at each meteorology station. Under the assumption of relatively smooth scale height changes within a certainty scale, the spatial distribution of H can be obtained by inverse distance weighted (IDW) interpolation method, and the $\sigma_{a,0}$ at each pixel can be calculated by Equation (1).

### 3.2. Relativity Correction

Based on the Mie theory, the extinction coefficient is proportional to the PM concentrations in the ambient air and can be expressed using the following equation [26,28]:

(3) $$\sigma_{a}\left(\lambda \right)=\frac{3\cdot \langle Q_{\mathrm{ext}}\rangle }{4\cdot r_{\mathrm{eff}}\cdot \rho }\cdot {\mathrm{PM}}_{x}$$

where $\langle Q_{\mathrm{ext}}\rangle$ is the size-distribution-integrated extinction efficiency, which is closely related to the aerosol composition and particle spectrum distribution [34]. $r_{\mathrm{eff}}$ is the effective radius, and ρ is the averaged mass density of the particles, which are related to RH [39,40]. ${\mathrm{PM}}_{x}$ is the mass concentration of PM. The parameters are affected by the environmental humidity due to the existence of a large number of hygroscopic components in particles.

Wang et al. [10] defined the average mass extinction efficiency ($E_{\mathrm{ext}}$) for an aerosol as the ratio of $\sigma_{a}\left(\lambda \right)$ to the ${\mathrm{PM}}_{x}$ concentrations. Hand and Malm [41] summarized that $E_{\mathrm{ext}}$ could be related to RH and expressed as a function of RH. Meanwhile, assuming that the chemical composition and aerosol distribution of aerosols would change little during a certain period, Liu [40] proposed that $E_{\mathrm{ext}}$ can be regarded as a function of ambient RH, and $E_{\mathrm{ext}}\left(\mathrm{RH}\right)$ can be translated to Equation (4):

(4) $$E_{\mathrm{ext}}\left(\mathrm{RH}\right)=\frac{\sigma_{a}\left(\lambda \right)}{{\mathrm{PM}}_{x}}=\frac{3\cdot \langle Q_{\mathrm{ext}}\rangle }{4\cdot r_{\mathrm{eff}}\cdot \rho }.$$

Importantly, $\sigma_{a}$ is the integration of the extinction coefficients of aerosol, but the PM_2.5_ concentrations were used to calculate the average mass extinction efficiency in this paper, which overestimated the ability of extinction and could lead to some uncertainty. Therefore, the average mass extinction efficiency describes the mean state of the extinction ability of particles for different properties.

To obtain the “dry” extinction coefficient, many studies have investigated the hygroscopic properties of aerosol particles [10,40,42,43,44]. An aerosol particle is influenced by water vapor in the atmosphere via a process called aerosol hygroscopic growth [28,45]. The aerosol hygroscopic growth factor f(RH) is defined as the ratio of aerosol extinction in ambient humidity to “dry” aerosol extinction under relatively dry conditions (RH less than 30%). In this study, the f(RH) can be described by Equation (5) [17,27,44,46,47,48,49].

(5) $$f\left(\mathrm{RH}\right)=\frac{\sigma_{a}\left(\lambda \right)}{\sigma_{\mathrm{dry}}\left(\lambda \right)}=\frac{E_{\mathrm{ext}}\left(\mathrm{RH}\right)}{E_{\mathrm{dry}}}=a+b\times {\left(\frac{\mathrm{RH}}{100}\right)}^{c}$$

where $\sigma_{\mathrm{dry}}$ is the extinction coefficient when RH is set below 30%. In this study, RH represents the relative humidity of the atmosphere, and a, b, and c are the parameters of f(RH) and can be obtained by fitting. Assuming that RH varies smoothly at a certain scale, the spatial map of RH can be obtained by IDW interpolation method from metrological sites. We assumed that the f(RH) demonstrates little change at a certain scale; hence, each pixel can find a nearest f(RH) to calculate PM_2.5_ concentrations.

(6) $${\mathrm{PM}}_{2.5}=\frac{\sigma_{a}(\lambda )}{E_{\mathrm{ext}}\left(\mathrm{RH}\right)}=\frac{\frac{\mathrm{AOD}}{H}}{\left(a+b\times {\left(\frac{\mathrm{RH}}{100}\right)}^{c}\right)\times E_{\mathrm{dry}}}$$

## 4. Results and Discussion

The physical and chemical properties of particles have large spatial and temporal variations; therefore, the capacity of hygroscopic growth differs. In this study, we selected three PM_2.5_ sites with different sources of pollution, which are in Xingtai-Nanhe, Qinhuangdao-Changli, and Zhangjiakou-Huaian (as indicated in Figure 1 by blue points), to analyze aerosol hygroscopic growth. The pollutants of Xingtai, which is one of the most polluted cities in China and is located to the east of Taihang Mountain, mainly come from anthropogenic activities and industry. Qinhuangdao is near the Bohai Sea, and sea salt represents part of pollutants. Taihang Mountain and Yanshan block pollutant transmission to Zhangjiakou, and the air quality of Zhangjiakou is relatively good. Therefore, the three sites can reflect spatial differences in aerosol hygroscopic growth.

### 4.1. Descriptive Statistics

**Figure 2:**
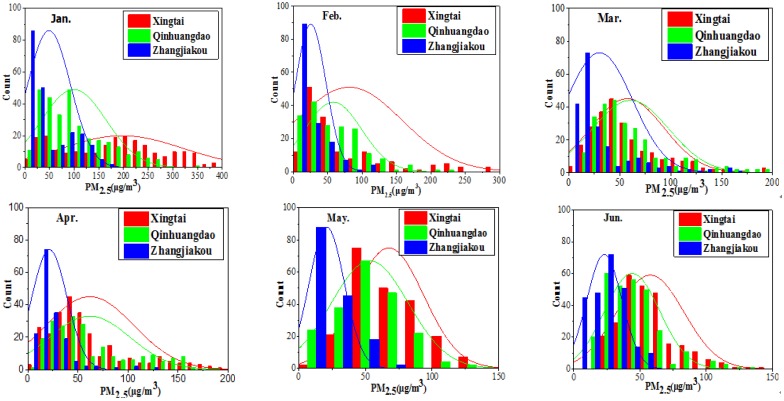
Histogram statistics of PM_2.5_ concentrations from Jan. to Jun. 2017 in Xingtai-Nanhe, Qinhuangdao-Changli, and Zhangjiakou-Huaian, respectively.

The summary statistics of factors (PM_2.5_, VIS, and RH) are listed in Table 1, and histograms of PM_2.5_ are presented in Figure 2. According to Table 1, the mean, median, and standard deviation (std) of the PM_2.5_ concentrations in Xingtai-Nanhe are 86.96 μg/m^3^, 57 μg/m^3^, and 81.93 μg/m^3^, respectively, indicating that the PM_2.5_ concentrations demonstrated a large change over the study period. Compared to Xingtai-Nanhe, Qinhuangdao-Changli has significantly low PM_2.5_ concentrations (mean, median, and std are 64.32 μg/m^3^, 51.50 μg/m^3^, and 46.53 μg/m^3^, respectively). Zhangjiakou-Huaian has the best air quality of the three sites, with a mean value of 29.34 μg/m^3^. The visibility of Qinhuangdao-Changli, with a mean value of 13.38 km, is relatively low compared to that of the other two sites due to the influence of water vapor from the sea. Compared to Qinhuangdao-Changli, Xingtai-Nanhe, and Zhangjiakou-Huaian have improved visibility (22.14 and 23.15 km, respectively). The study area is located in North China, which has a low-rainfall period and accordingly low RH in spring and winter [50] (RH of 57.21%, 59.19%, and 44.54% in Xingtai-Nanhe, Qinhuangdao-Changli, and Zhangjiakou-Huaian, respectively).

According to Figure 2, the means of the monthly and maximum PM_2.5_ concentrations of the three sites gradually decreased from January to June 2017. This decrease can be attributed to the following reason: a large amount of pollutants were discharged into the atmosphere during coal heating in January and February, and the static stability weather conditions, such as the relatively low boundary layer height and rainfall, were not conducive to pollutant diffusion, resulting in higher PM_2.5_ concentrations. However, in spring and summer, the PM_2.5_ concentrations were relatively low because the atmosphere was relatively active, thus favoring pollutant diffusion, and coal heating was stopped, thus reducing the source of pollutants. The air quality of Xingtai-Nanhe was more likely to be “heavily” and “severely” polluted in January than in other months, and the maximum PM_2.5_ value reached 573 μg/m^3^. The number of high PM_2.5_ concentrations decreased after January, with a maximum monthly value of only 219 μg/m^3^ in February. There were more good air quality cases in May and June than in other months. In general, the PM_2.5_ concentrations were lower in Qinhuangdao-Changli than in Xingtai-Nanhe, and the highest PM_2.5_ value was 359 μg/m^3^. However, the air quality in Qinhuangdao-Changli was most commonly “moderately” polluted in April. The air quality of Zhangjiakou-Huaian was better than that of the other sites, and the PM_2.5_ concentrations were generally less than 50 μg/m^3^, except for in January.

### 4.2. $E_{ext}\left(RH\right)$ Fitting and f(RH) Analysis

**Figure 3:**
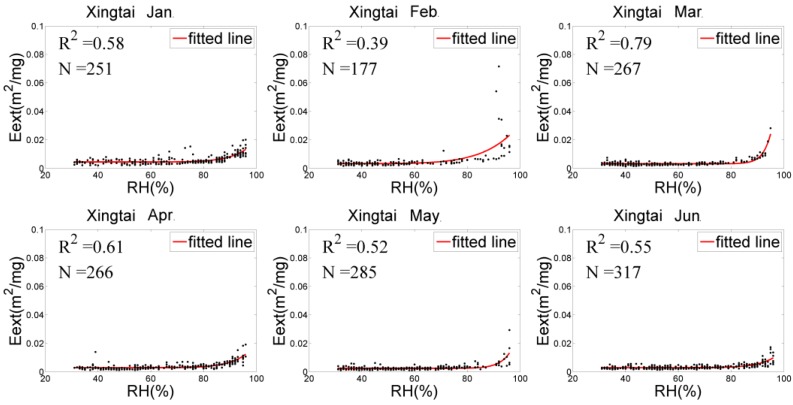
$E_{\mathrm{ext}}\left(\mathrm{RH}\right)$ fitting at Xingtai-Nanhe.

**Figure 4:**
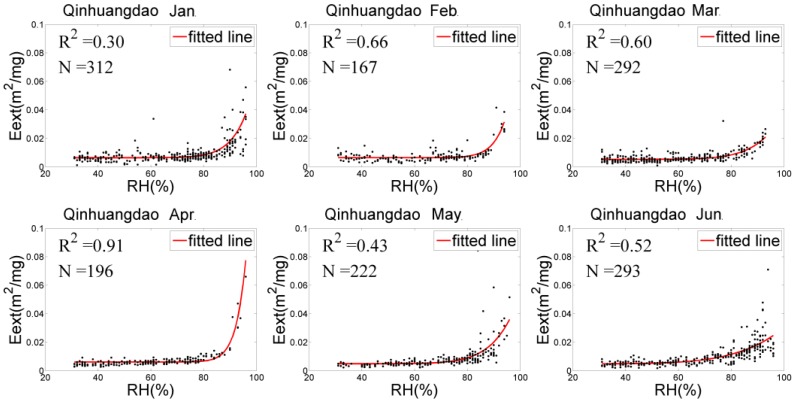
$E_{\mathrm{ext}}\left(\mathrm{RH}\right)$ fitting at Qinhuangdao-Changli.

**Figure 5:**
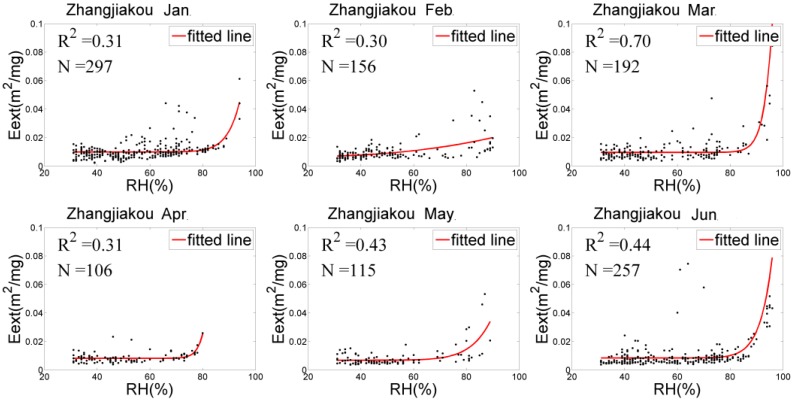
$E_{\mathrm{ext}}\left(\mathrm{RH}\right)$ fitting at Zhangjiakou-Huaian.

The relationships between $E_{\mathrm{ext}}\left(\mathrm{RH}\right)$ and RH of the three stations were adequately fitted by Equation (5), as shown in Figure 3, Figure 4 and Figure 5. In general, $E_{\mathrm{ext}}\left(\mathrm{RH}\right)$ grows slowly when RH is low and increases rapidly under higher RH. However, there were differences in RH among the three sites, showing the following: (1) The scatter distribution had the most concentrations and the best fitting ability at Xingtai-Nanhe, and the R^2^ was higher than 0.5 (except for in February). At Qinhuangdao-Changli, the fitting ability was the worst in January and the best in April (R^2^ of 0.3 and 0.9, respectively). The hygroscopic growth capacity was stronger at Qinhuangdao-Changli than at the other two sites because Qinhuangdao-Changli is close to the coast, the aerosol compositions of this coastal site contain salt particles, and the $E_{\mathrm{ext}}\left(\mathrm{RH}\right)$ rapidly grows with RH when RH is more than 70%. The fitting result in Zhangjiakou-Huaian was not ideal, and the R^2^ was generally less than 0.5 in each month. (2) At Xingtai-Nanhe, the fitting curve from January to June was relatively flat when RH was less than 90% and increases slightly when RH was more than 90%, which shows that the capacity of hygroscopic growth was weak. Compared with the fitting curve of Xingtai-Nanhe, the Qinhuangdao-Changli curve increased more rapidly when RH exceeded 90%. The data from both March and May presented obvious hygroscopic behavior, and a deliquescent point with an RH of approximately 90% occurred in April, while a hygroscopic and deliquescent phenomenon occurred in January and June. At Zhangjiakou-Huaian, the humidographs from January to June had flat growths at a medium RH (40–80%), with sharp increases under high RH (>80%) in March and June, which shows that both hygroscopic and deliquescent behaviors occurred simultaneously. Therefore, there were significant differences in the physical and chemical characteristics of aerosols in the three regions. (3) There was little variation in the fitting curve of Xingtai-Nanhe, which indicates that the aerosol sources and environmental conditions in the area varied little from month to month. The capacity of hygroscopic growth had a high monthly variation at Qinhuangdao-Changli, indicating that the site experienced large environmental changes or had complex aerosol sources because of aerosol transmission from other areas. Although the existence of outliers within the fitting results can generate an uncertainty for humidity correction, the results were able to reflect the average variation of $E_{\mathrm{ext}}\left(\mathrm{RH}\right)$ with RH. It is helpful to correct the influence of humidity to obtain near-surface particle concentrations.

The monthly and half-year mean humidification factor values at RH = 80%, f(80%) were calculated for the three sites by Equation (5), as listed in Table 2. The f(80%) value in Qinhuangdao-Changli was generally higher than those of the other two sites (except in February), with a range from 1.39 to 2.39, which shows hygroscopic growth has a stronger capacity with RH. The mainly reason for this trend is that aerosol particles near coastal sites have a relatively high proportion of sea salt components, and their overall hygroscopic growth ability is the strongest. The proportion of sea salt particles at inland sites such as Zhangjiakou-Huaian and Xingtai-Nanhe is small, and the proportion of black carbon aerosols is large; thus, hygroscopic growth at inland areas is relatively weak. However, the capacity of hygroscopic growth can have great variations over different months at the same site. For example, at Xingtai-Nanhe, the f(80%) value was the highest in February and the lowest in March, with values of 2.23 and 1.01, respectively. At Qinhuangdao-Changli, the highest (lowest) value of f(80%) was 2.39 (1.39) in May (February). At Zhangjiakou-Huaian, the highest (lowest) value of f(80%) was 4.34 (1.35) in February (June). According to the above analysis, the hygroscopic growth of particles in different regions and at different times varies greatly with the RH. Therefore, it is necessary to perform hygroscopic correction at each site in this paper in order to improve the estimation accuracy of PM_2.5_ concentrations.

### 4.3. The Results of PM_2.5_ Estimation

#### 4.3.1. Vertical-Humidity Correction on AOD

**Figure 6:**
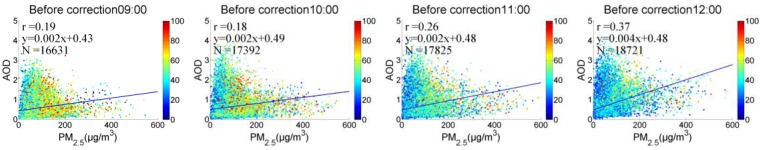

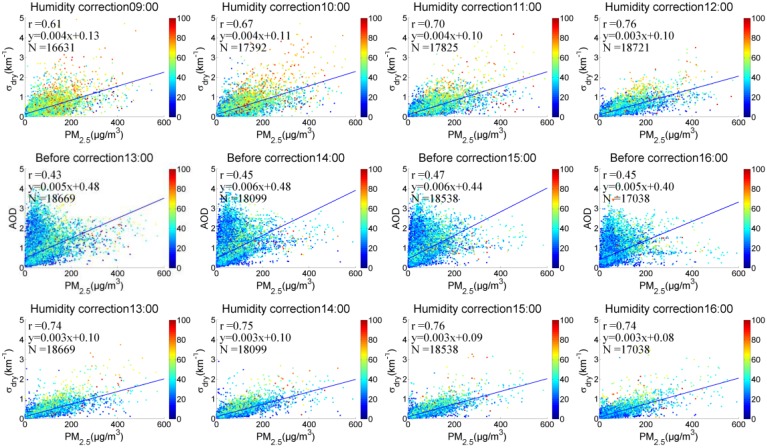
Scatterplots of both AOD with PM_2.5_ and $\sigma_{\mathrm{dry}}$ with PM_2.5_ for different hours (09:00–16:00 local times) in Hebei (colorbar represents RH).

According to the matching data of the environmental monitoring stations and meteorological stations in Hebei province, vertical and humidity corrections were made to each site to estimate the PM_2.5_ concentrations. Both the scatterplots of PM_2.5_ vs. AOD and PM_2.5_ vs. the “dry” extinction coefficient ($\sigma_{\mathrm{dry}}$) are shown in Figure 6 (colorbar indicates RH). The first and third rows represent the scatterplots between AHI AOD and PM_2.5_ from 09:00 to 16:00, and the second and fourth rows represent $\sigma_{\mathrm{dry}}$ and PM_2.5_. The scatter distributions of AOD and PM_2.5_ are relatively discrete, and their correlations are low (the lowest r is 0.18 at 10:00, and the highest r is only 0.47 at 15:00). Compared to the poor correlation between AOD and PM_2.5_, a better, relatively high correlation was obtained by vertical-humidity correction between $\sigma_{\mathrm{dry}}$ and PM_2.5_, with the hour correlation r increasing from 0.19–0.47 to 0.61–0.76.

#### 4.3.2. PM_2.5_ Estimation Validation

**Figure 7:**
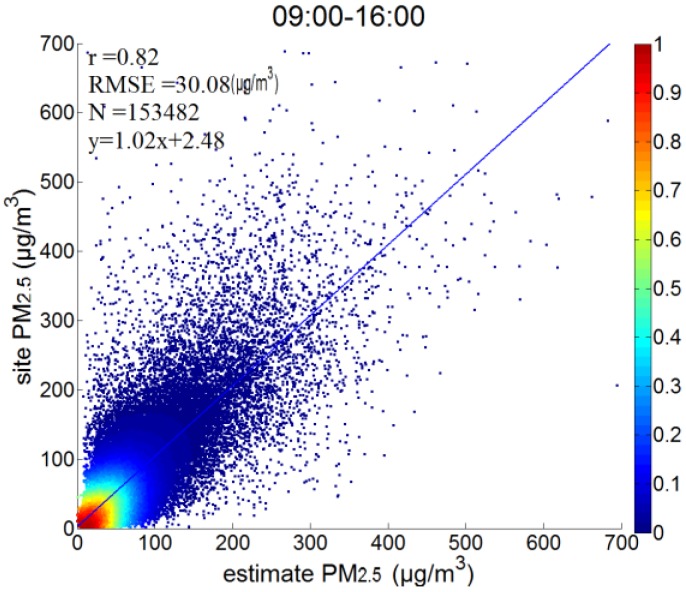
Scatterplots of satellite-retrieved and ground-measured PM_2.5_ from January to June 2017 in Hebei.

The relationship between the near-surface “dry” extinction coefficient and PM_2.5_ concentrations improved after vertical-humidity correction; therefore, the PM_2.5_ concentrations were calculated by Equation (6). The satellite-estimated PM_2.5_ and all ground-measured data from January to June 2017, in which there are a total of 153,482 points, are shown in Figure 7. According to the fitting results, the correlation was relatively high (r = 0.82), the root mean square error (RMSE) was 30.08 μg/m^3^, the slope was close to 1, and the intercept was 2.48. The accuracy of PM_2.5_ estimation was verified on a daily, monthly, and by site.

(1) Monthly PM_2.5_ Validation

**Figure 8:**
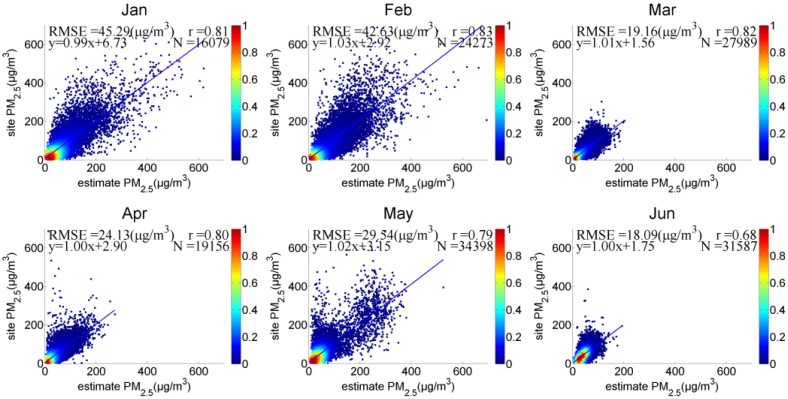
Monthly PM_2.5_ of satellite-retrieved and ground-measured data in Hebei.

To analyze the accuracy of PM_2.5_ estimation at the monthly scale, the scatterplot for each month is shown in Figure 8. The r value of each month was approximately 0.8 (except for June, r is 0.68), and the slope was between 0.99 and 1.03. Hebei was “heavily” polluted and had high PM_2.5_ concentrations in January and February. After vertical-humidity correction, the r value increased (r = 0.81 and 0.82 in January and February, respectively), but the dispersion of some scattered points was relatively large, and the RMSE increased (45.29 and 42.63 μg/m^3^ in January and February, respectively). The air was relatively dry in winter, and the capacity of hygroscopic growth was weak; hence, humidity correction had no obvious effect at some sites. The r values in March, April, and May were 0.8 ± 0.1, and the PM_2.5_ concentrations and RMSE values (RMSEs of 19.16, 24.13, and 29.54 μg/m^3^, respectively) were lower than those in both January and February. The points were relatively concentrated in March and April, but some high values were dispersed in May. The RMSEs were the lowest in June (RMSE = 18.09 μg/m^3^), and the slope was equal to 1.

(2) Daily PM_2.5_ Validation

**Figure 9:**
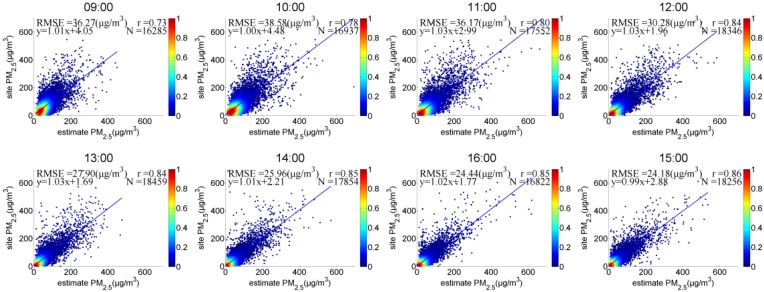
Hourly PM_2.5_ of satellite-retrieved and ground-measured PM_2.5_ data in Hebei.

Figure 9 presents hourly daytime PM_2.5_ scatter plots from January to June 2017. The correlation increased from 09:00 to 16:00, and the r increased from 0.73 to 0.86, with the maximum (minimum) RMSE of 38.58 μg/m^3^ (24.18 μg/m^3^) at 10 a.m. (15 p.m.) and higher PM_2.5_ concentrations in morning than in afternoon, which might be attributed to the following two factors. (1) In the morning, the solar azimuth and water vapor level are relatively high, and sunlight reaches the ground after a longer path through the atmosphere, which can affect the accuracy of AOD retrieval. (2) The higher RH can cause deliquescent behaviour, which has an influence on the humidity correction. In June 2017, the correlation was relatively low, with r = 0.68, which might have occurred because the atmosphere was more active, with higher wind speeds and increases in rainfall. Consequently, the physical and chemical properties of particulates in local areas were more complex and varied greatly.

(3) Site-Based PM_2.5_ Validation

**Figure 10:**
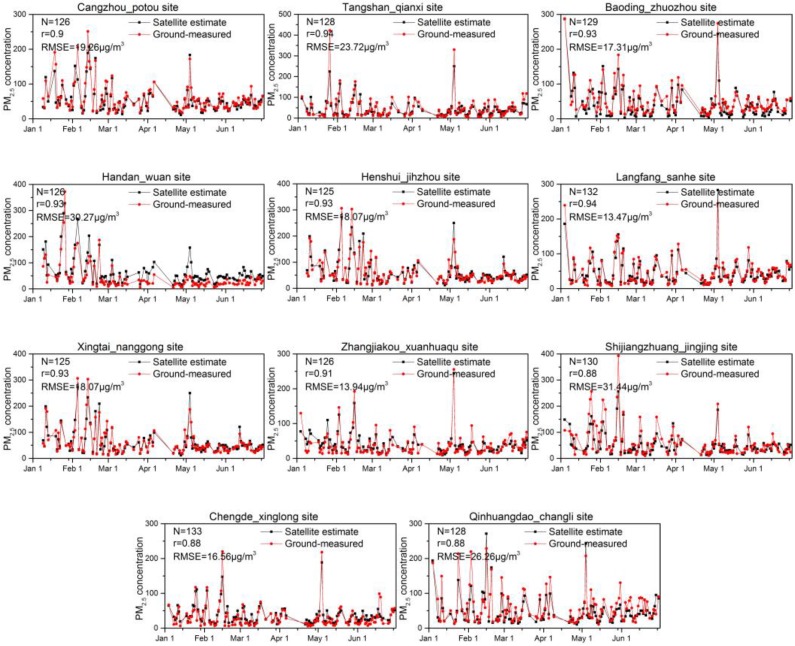
Daily averaged PM_2.5_ of satellite-retrieved and ground-measured PM_2.5_ data at Baoding-Zhuozhou, Cangzhou-Potou, Tangshan-Qianxi, Handan-Wuan, Hengshui-Jinzhou, Langfang-Sanhe, Xingtai-Nanggong, Zhangjiakou-Xuanhuaqu, Shijiazhaung-Jingjing, Chengde-Xinglong, and Qinhuangdao-Changli.

**Figure 11:**
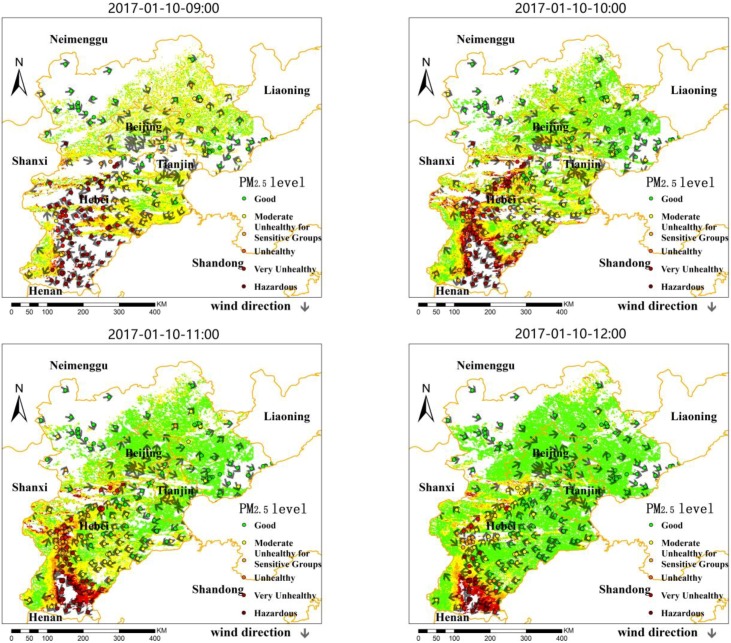

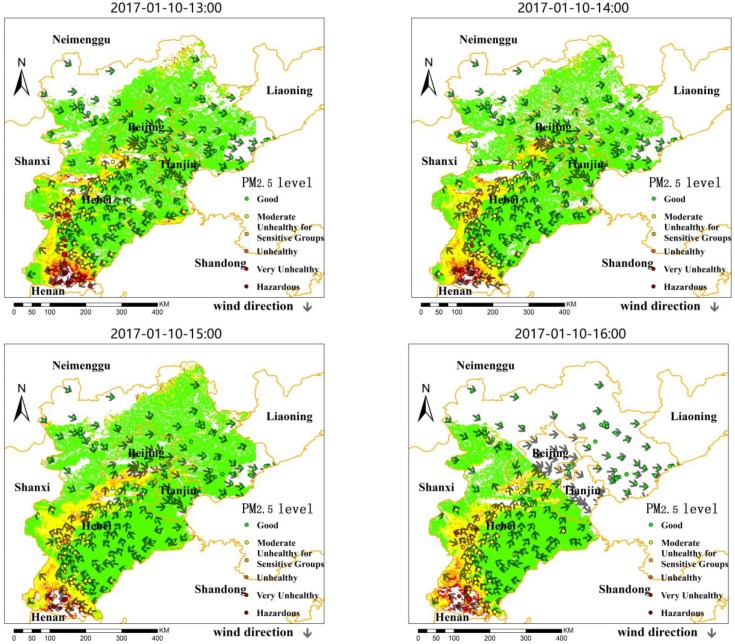
Hourly PM_2.5_ concentrations of satellite-retrieved and ground-measured from 09:00 to 16:00 on 10 January 2017, in Hebei.

To evaluate the accuracy of the PM_2.5_ vertical-humidity correction at each site, we selected 11 sites from different cities in Hebei and respectively drew a time series diagram of the daily mean PM_2.5_ concentrations, as shown in Figure 10. According to the ground-measured PM_2.5_, in January and February 2017, there was “heavy” pollution weather (PM_2.5_ > 150 μg/m^3^) at Baoding, Changzhou, Handan, Hengshui, Xingtai, and Shijiazhuang, where the air quality was very poor. There were low PM_2.5_ concentrations at Zhangjiakou, Tangshan, and Chengde, where there were few pollutants from industry and vehicle emissions. At all sites, except for on 4, May 2017 (PM_2.5_ > 200 μg/m^3^), the mean daily PM_2.5_ concentrations from March to June were generally less than 100 μg/m^3^, which shows good air quality. The decrease in PM_2.5_ was mainly related to meteorological conditions and decreases in pollution from coal heating. According to Figure 11, the correlation between ground-measured PM_2.5_ and satellite estimation was relatively high overall. The r values were generally ±0.9, and the RMSEs were between 13.94 and 31.44 μg/m^3^. However, PM_2.5_ concentrations were overestimated (underestimated) when the PM_2.5_ concentrations was low (high). Interestingly, satellite estimation commonly underestimated the PM_2.5_ of Qinghuandao-Changli, as the PM_2.5_ of this site has complex physical and chemical characteristics, but the particle component were not considered in this paper, which may have a certain influence on the accuracy of PM_2.5_ estimation.

#### 4.3.3. Hourly Patterns of PM_2.5_ Concentration

Assuming that particle composition and weather condition are basically stable, the f(RH) of each pixel can be matched by the nearest neighbour searching principle in order to estimate the spatial distribution of PM_2.5_. The larger daily variation of PM_2.5_ in this section is selected to analyze the hourly change process on 10 January 2017, as shown in Figure 11 (the classification standard adopts the ambient air quality standard of China). Except for a few stations with underestimated results, the satellite-estimated PM_2.5_ concentrations at the air quality level agreed well with the ground-based measurements, so the satellite data can clearly reflect the spatial distribution of pollution. Clouds covered a larger area (blank area) at 09:00 over Hebei, but the ground-measured PM_2.5_ concentrations show that the air quality was poor, and Baoding, Shijiazhuang, Hengshui, and Xingtai were especially heavily polluted. From 09:00 to 12:00, the wind direction changed from an east wind from the Bohai Sea to Hengshui to mainly northerly winds and southerly winds; accordingly, the pollution over Cangzhou, Langfang, and Hengshui migrated to the southern regions, leading to high PM_2.5_ concentrations in Xingtai and Handang. The south wind predominated over Shijiazhuang and Baoding at 13:00, and pollutants migrated from Handan to Xingtai, Shijiazhuang, and Baoding along the Taihang Mountain range, forming an obvious pollution zone. Therefore, the high spatial-temporal resolution PM_2.5_ data can continuously and intuitively reflect the characteristics of regional pollutants (such as diffusion and accumulation), which is of great significance for the assessment of regional air quality.

## 5. Conclusions

This study analyzed the hygroscopic growth characteristics of particulate matter in different regions of Hubei province, improved the estimation method of PM_2.5_, obtained high spatial-temporal resolution AHI AOD data to estimate hourly PM_2.5_ concentrations, and evaluated the estimated accuracy. The main conclusions include the following:

1. Three sites located in different regions of Hebei province were selected to analyze the capacity of hygroscopic growth. Qinhuangdao-Changli, with a sea salt pollutant component, has the highest hygroscopic growth ability, while Zhangjiakou-Huaian has the second highest hygroscopic growth ability, and Xingtai-Nanhe, with a high black carbon pollutant component, has the lowest hygroscopic growth ability; these results indicate that the physicochemical characteristics of the particles in different regions are inconsistent. Thus, vertical-humidity correction is helpful to improve the accuracy of PM_2.5_ estimation in different regions.
2. Compared to the relationship between AOD and PM_2.5_, the relationship between $\sigma_{a,\mathrm{dry}}$ and PM_2.5_ significantly improved, with the coefficient r increasing from 0.19–0.47 to 0.61–0.76. The accuracy of PM_2.5_ estimation is verified at the hourly, daily, and monthly scales, respectively. The hourly PM_2.5_ estimation is relatively high r (0.8 ± 0.07), with a low RMSE (30.4 ± 5.5 μg/m^3^), and the accuracy in the afternoon (13:00 to 16:00) is higher than that in the morning (09:00 to 12:00). In a comparison of the daily average PM_2.5_ concentrations at 11 sites, the r value is approximately 0.9, and the RMSE is between 13.94 and 31.44 μg/m^3^. The result suggested that the new method in this study is useful to improve the accuracy of PM_2.5_ estimation.
3. The spatial distribution of PM_2.5_ concentrations from 09:00 to 16:00 is estimated for 10 January 2017, and the process of pollution accumulation and dissipation is clearly presented over space and time. This type of estimation is conducive to the evaluation and control of air quality.

The use of the vertical-humidity method to estimate the spatial distribution of PM_2.5_ yielded results with a relatively high accuracy, but obtaining the hygroscopic growth factor far from the ground monitoring site can impact the estimation accuracy when the meteorological conditions change greatly. The particulate composition, which affects the accuracy of PM_2.5_ estimation, was not considered in this study. Therefore, obtaining more ground-based data and research on the composition of particles will help improve the PM_2.5_ inversion accuracy in future research.

## Figures and Tables

**Table 1:** Statistical hourly PM_2.5_ data from January to June 2017.

Variable	Value	Xingtai-Nanhe	Qinhuangdao-Changli	Zhangjiakou-Huaian
PM_2.5_ (μg/m^3^)	mean	86.96	64.32	29.34
	median	57.00	51.50	19.00
	std	81.93	46.53	28.88
VIS (km)	mean	22.14	13.38	23.15
	median	25.56	11.55	24.26
	std	12.60	8.62	11.08
RH (%)	mean	57.21	59.19	44.54
	median	56.00	66.00	40.00
	std	24.14	24.92	24.52

**Table 2:** Hygroscopic growth ability of f(80%) at Xingtai-Nanhe, Qinhuangdao-Changli, and Zhangjiankou-Huaian.

Month	Xingtai-Nanhe	Qinhuangdao-Changli	Zhangjiakou-Huaian
January	1.13	1.61	1.78
February	2.23	1.39	4.34
March	1.01	1.81	2.13
April	1.06	2.08	1.96
May	1.18	2.39	2.23
June	1.20	2.03	1.35
Half-year	1.32	1.84	1.28
